# Driving Anger, Aberrant Driving Behaviors, and Road Crash Risk: Testing of a Mediated Model

**DOI:** 10.3390/ijerph16030297

**Published:** 2019-01-22

**Authors:** Tingru Zhang, Alan H. S. Chan, Hongjun Xue, Xiaoyan Zhang, Da Tao

**Affiliations:** 1 Institute of Human Factors and Ergonomics, College of Mechatronics and Control Engineering, Shenzhen University, Shenzhen 518060, China; zhangtr@szu.edu.cn (T.Z.); taoda@szu.edu.cn (D.T.); 2 Department of Systems Engineering and Engineering Management, City University of Hong Kong, Hong Kong, China; alan.chan@cityu.edu.hk; 3 School of Aeronautics, Northwestern Polytechnical University, Xi’an 710072, China; xuehj@nwpu.edu.cn; 4 Key laboratory of Optoelectronic Devices and Systems of Ministry of Education and Guangdong Province, Shenzhen University, Shenzhen 518060, China

**Keywords:** driving anger, aberrant driving behaviors, road crash risk, mediated model

## Abstract

With the dramatic increase in motorization, road traffic crashes have become the leading cause of death in China. To reduce the losses associated with road safety problems, it is important to understand the risk factors contributing to the high crash rate among Chinese drivers. This study investigated how driving anger and aberrant driving behaviors are related to crash risk by proposing and testing one mediated model. In this model, the effects of driving anger on road crash risk were mediated by aberrant driving behaviors. However, unlike previous studies, instead of using the overall scale scores, the subscales of driving anger and aberrant driving behaviors were used to establish the mediated model in this study. To test the validity of this model, an Internet-based questionnaire, which included various measures of driving anger, aberrant driving, and road crash history, was completed by a sample of 1974 Chinese drivers. The results showed that the model fitted the data very well and aberrant driving behaviors fully mediated the effects of driving anger on road crash risk. Findings from the present study are useful for the development of countermeasures to reduce road traffic crashes in China.

## 1. Introduction

The number of motorized vehicles in China has increased 10-fold in the past 15 years, from about 16 million in 2000 to 163 million in 2015 [1]. Along with this dramatic increase in motorization, road safety has become a major public health problem [2]. In 2017, road fatalities were as high as 45.9 per 100,000 persons and 305.0 per 100,000 motor vehicles in China. [1]. The problem of traffic crashes has attracted much attention from driving safety researchers in recent years, and many related studies have been reported [2,3]. However, the risk factors associated with the high rate of traffic accidents in China are still unclear. According to some recent studies [4,5,6], Chinese drivers are more likely to experience road rage while driving due to conflicts among drivers and traffic congestion in Chinese urban cities [7]. It is very likely that driving anger and associated aberrant driving behaviors could be significant predictors of road crash risk among Chinese drivers.

### 1.1. Driving Anger, Aberrant Driving Behaviors, and Road Crash Risk

Anger refers to a psychological emotional state characterized by feelings of annoyance, fury, or rage. It is generally accompanied by muscular tension and arousal of the autonomic nervous system [8]. Driving anger is one of the most frequently experienced emotions on the road [9]. In driving safety literature, the 14-item Driving Anger Scale (DAS) [10] has been widely used to measure the trait of driving anger, which is the tendency of drivers to become angry while driving. This instrument requires respondents to rate the amount of anger experienced when encountering 14 potentially anger-provoking scenarios on a 5-point scale. In studying the impacts of driving anger on crash-related conditions (e.g., traffic tickets, losing concentration, near misses, and traffic crashes), Deffenbacher et al. [11] found that drivers with a higher level of driving anger (DAS > 3.7) were twice as likely to crash in simulated driving compared with those with a lower anger level (DAS < 3.0). Dahlen et al. [12] showed that driving anger was a positive predictor of loss of concentration and near misses on the road, but it was not significantly associated with minor or major accidents. In a recent questionnaire-based study, Sullman and Stephens [13] showed that driving anger significantly contributed to the prediction of near misses, but not to traffic tickets, losing concentration, or road crashes. These inconsistent findings, together with the fact that past studies were carried out in Western countries, raise some doubts as to whether driving anger is a relevant factor for road crash risk in China and what the working mechanisms are behind the anger–crash relationship.

Compared with driving anger, a driver’s aberrant driving behaviors seem to be stronger and more direct predictors of road crash risk. According to Qu et al. [14], risky and aggressive driving behaviors, such as speeding or running red lights, accounted for approximately 94.4% of all traffic deaths in China. In road safety studies, the Driver Behavior Questionnaire (DBQ) has proven to be a valid measurement scale to examine drivers’ self-reported aberrant behaviors [15,16]. When initially proposed by Reason et al. [15], the DBQ contained 50 items, which were classified into three subscales, namely, violations, errors, and lapses, to capture different aspects of driving behaviors. In subsequent applications, the contents of the DBQ have been revised to fit the actual needs of different studies and additional subscales, such as emotional violations [17] and interpersonal violations [18], have been reported. However, the psychological distinction between violations and errors is particularly robust and these two subscales have been reported in almost all related studies (e.g., [17,19,20]). Crash risk is related to both the tendency to commit violations [18,21] and the tendency to make errors [22]. In a comprehensive review of the DBQ as a predictor of traffic crashes, De Winter and Dodou [23] showed that violations and errors have comparable strengths in predicting self-reported crashes.

Driving anger and aberrant driving behaviors have been shown to be closely related [24]. It has been demonstrated that anger interferes with human cognitive processes, such as attention [25] and judgment [26], making angered individuals exhibit excessive optimism and reduced risk perception. As a result, drivers who have reported a greater level of anger are more likely to commit violations (e.g., tailgating and speeding) on the road [12,13,27,28]. Different relations between driving anger and driving errors have been reported. A majority of studies have found a positive relation [28,29], while a few have reported a nonsignificant association [30]. However, recent evidence suggests that the anger–aberration relationship could be more complex than a simple positive association [7]. Zhang et al. [7] found that three types of driving anger (hostile gesture anger, arrival-blocking anger, and safety-blocking anger), differing in their goal-blocking nature, can be measured by the 14-item DAS. In particular, hostile gesture anger refers to anger triggered by hostile gestures or language; arrival-blocking anger refers to anger triggered by events that slowed the movement of the driver; and safety-blocking anger refers to anger triggered by events that might threaten the safety of the driver. More importantly, these three types of driving anger showed dissimilar associations with driving aberrations. All these recent findings suggest that it is necessary to investigate anger–aberration relations on their subscale levels.

### 1.2. The Mediated Model

Previous research has indicated strong relationships among driving anger, aberrant behaviors, and road crash risk. However, only a few studies have tried to integrate them into one single model [31,32,33]. In a review of the causes of road crashes, Elander et al. [31] proposed a mediated model to describe the relations among personality factors, driving behaviors, and road crash risk. In this mediated model, personality factors (e.g., driving anger) have an indirect impact on crash risk through their influences on driving behaviors (the mediator). The validity of such a mediated model has been investigated in two questionnaire studies [32,33]. A study conducted on Norwegian drivers [32] showed that risky driving behaviors (e.g., speeding) partially mediated the effects of driving anger on road crash involvement. However, in a study on Turkish professional drivers [33], the effects of driving anger on road crash risk were found to be mediated by dysfunctional drinking behaviors rather than aberrant driving behaviors.

There are at least three reasons that may explain these inconsistent findings. The first is that both DAS and DBQ, used to measure driving anger and driving aberrations, respectively, have been demonstrated to contain subscales differing in psychological characteristics. As a result, the relationships established using the overall scores of DAS and DBQ may have obscured the real associations at the subscale level. For example, Zhang et al. [7] recently found that safety-blocking anger was negatively associated with deliberate driving violations, though the overall anger–aberration relation was positive. As a result, for a more accurate understanding of the relations of driving anger, aberrant driving, and road crash risk, the subscales, rather than an overall score, should be used to establish the model. Second, road crash risk was measured in different ways in two studies. Sümer [33] only used the number of crashes, while Iversen and Rundmo [32] included both crashes and near misses as measures of crash risk. According to Dahlen et al. [12], for a comprehensive representation of crash risk, different crash-related conditions should be measured. Finally, the inconsistent findings of the two mediated models mentioned above may be due to a lack of control of the influence of demographic variables in the two studies. Since there has been evidence that age and gender, as well as driving experience, are significantly related to crash risk [34,35,36], the real associations of driving anger, aberration, and crash risk may have been masked or biased by the confounding effects of these demographic variables.

**Figure 1:**
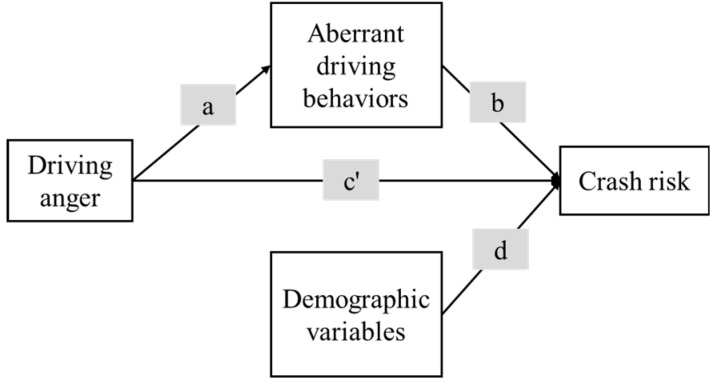
Framework of the mediated model proposed and tested in this study. It is hypothesized that the effects of driving anger on crash risk would be fully mediated by aberrant driving behaviors (i.e., a*b ≠ 0, and *c*’ = 0).

To overcome the three abovementioned limitations in previous works, the present study aimed to establish more accurate relations between driving anger, aberrant driving behaviors, and road crash risk by proposing and verifying the validity of a mediated model. The framework of the proposed mediated model is shown in Figure 1. Specifically, the 14-item DAS was used to measure driving anger and the DBQ was used to record aberrant driving behaviors. The subscales of DAS and DBQ, rather than overall scores, were applied in the model. Information about four crash-related conditions (traffic tickets, losing concentration, near misses, and traffic crashes) was collected to represent the latent variable crash risk. Path *a* represents the effect of driving anger on aberrant driving behaviors (the mediator); path *b* represents the effect of the mediator on the crash risk; and path *c* represents the direct effect of driving anger on crash risk. The total effect of driving anger on crash risk can be apportioned into its indirect effect (i.e., mediated effect, a*b) and direct effect (path *c’*). The mediated effect through aberrant driving behaviors is significant when the product of paths *a* and *b* is significantly different from zero [37,38]. The effects of demographic variables on crash risk are controlled for by including path *d* in the model [39]. It is hypothesized that the effects of driving anger on crash risk would be fully mediated by aberrant driving behaviors. That is, driving anger would have no direct influence on crash risk (*c’* = 0), but would directly affect aberrant driving behaviors, which, in turn, influence crash risk (a*b ≠ 0). The findings from this study provide a better understanding of the role of driving anger and aberrant driving in the causation of road traffic crashes. The results are also useful for the development of effective road safety countermeasures in China.

## 2. Materials and Methods 

The Internet has proven to be a valid tool for assessing driver behaviors in China [17]. The online questionnaire survey technique was used for collecting data in this study. Invitations to participate in the survey were posted on the Autohome Forum (www.autohome.com.cn). The forum has 10 million daily active users nationwide and is the biggest driver forum in China. Those interested were directed to complete the questionnaire published on Sojump (www.sojump.com), a professional online survey platform. Participants were required to first read the electronic consent form and only those who agreed to participate were directed to complete the questionnaire. The study was approved by the Institutional Review Board of Shenzhen University. The survey was open for two weeks and a total of 1974 valid responses were collected.

### 2.1. Structure of the Questionnaire

There were four sections in the questionnaire for measuring driving anger, aberrant driving behaviors, road crash risk, and demographic variables.

#### 2.1.1. DAS

The short, 14-item DAS, initially proposed by Deffenbacher et al. [10] and translated into Chinese by Zhang et al. [7], was used here to measure drivers’ anger level. This instrument contains 14 anger-provoking scenarios and respondents are required to rate the amount of anger experienced for each scenario on a 5-point scale (1 = not at all, 2 = a little, 3 = some, 4 = much, and 5 = very much). Both the original 14-item short version and the Chinese translated version have shown good internal consistency. However, Deffenbacher et al. [10] claimed that the short DAS was a one-factor structure while Zhang et al. [7] showed that a three-factor structure of DAS (hostile gesture, safety-blocking, and arrival-blocking) best fit the data.

#### 2.1.2. DBQ

The 22-item Chinese version of the DBQ, developed by Zhang et al. [7], was used here to examine self-reported aberrant driving behaviors. It contains 22 common aberrant driving behaviors (e.g., “*sound horn to indicate your annoyance to another road user*”) and respondents need to indicate how often each aberration occurred to them in the past 12 months on a scale between 0 (never) and 5 (nearly all the time). Zhang et al. [7] demonstrated that this Chinese version of the DBQ measured four types of aberrant behaviors, namely, emotional violation, maintaining progress violation, deliberate violation, and error. The four subscales were moderately correlated and all had acceptable internal consistency. 

#### 2.1.3. Road Crash Risk

In this section, participants were required to report the number of times they had encountered each of the four crash-related conditions (traffic tickets, losing concentration, near misses, and traffic crashes) in the past 12 months. A one-year period was chosen to correspond to the aberrant driving behavior recording period in the DBQ. In the questionnaire, a near miss was defined as an unplanned event that had the potential to cause, but did not actually result in, human injury, environmental or equipment damage, or an interruption to normal operation [40]. An accident was defined as an unplanned event that resulted in personal injury or property damage [40]. No definition for “losing concentration” was provided in the questionnaire, as there was no standard definition for this term.

#### 2.1.4. Demographic Variables

It has long been recognized that demographic variables, such as age, gender, and driving experience, are significantly related to crash involvements. In this section, participants were asked to report their age, gender, and years of active driving (“<3 years”, “3–5 years”, “6–10 years”, or “>10 years”). Driving experience was assessed with “years of active driving” instead of “years licensed” for two reasons. First, there may be inactive licensed drivers who rarely drive after obtaining a driving license. Second, some Chinese drivers begin to drive before obtaining a driving license [41].

### 2.2. Statistical Analysis

Principal component analysis (PCA) is a frequently used technique to reorganize a number of items from one measurement scale into a much smaller number of principal components (factors) while retaining as much information as possible. In this study, PCA with varimax rotation [42] was performed to analyze the factor structure of the DAS and DBQ. While a set of factors can be generated by PCA, only a few factors account for meaningful amounts of item variance. Two criteria are usually used to determine the factors that should be retained. First, according to the Kaiser criterion, a valid factor should have an eigenvalue of >1 [43]. Second, the factor should satisfy the internal consistency requirement by showing a Cronbach’s α > 0.60 [44]. An item would be grouped into the factor with which it had the highest correlation (i.e., factor loading). However, when the value of the highest correlation was less than 0.4 [45], the item was excluded from further analysis. PCA offered a method to investigate the factor structure of DAS from an exploratory analysis perspective, which was a supplement to past confirmatory factor analysis (CFA) studies [7,13].

Structural equation modeling (SEM) with AMOS software (IBM, Chicago, IL, USA) was used to test the goodness of fit of the mediated model and to calculate the path coefficients in the model. While there are no golden rules, Kline [46] has advocated reporting three indices for assessing model fit: The comparative fit index (CFI), the root mean square error of approximation (RMSEA), and the standardized root mean square residual (SRMSR). In this study, the model fit was examined with these three indices and a model was considered good when CFI > 0.95, RMSEA < 0.08, and SRMSR < 0.08 [47]. The direct, indirect, and total effects [48] in the model have also been reported.

## 3. Results

### 3.1. Descriptive Statistics

The participants had an average age of 32.61 years (*SD* = 6.29) and the majority (92.0%) of them were male drivers. Regarding driving experience, 44.9% of respondents were in the “<3 years” group, 20.7% in the “3–5 years” group, 22.7% in the “6–10 years” group, and the remaining 11.7% in the “>10 years” group.

Table 1 shows the mean ratings of DAS, DBQ instruments, and the mean reported frequency of the four crash-related conditions. The mean DAS score of 2.45 was comparable to that reported in one recent Chinese driver anger study (2.54 in Li et al. [49]). The mean DBQ score of 2.19 was higher than the score of 1.26 reported in a past Chinese driver behaviors study [17].

### 3.2. Psychometric Properties of DAS

The content and mean rating for each item of DAS is presented in Table 2. In this study, PCA, instead of CFA, was used to analyze the factor structure of the 14-item DAS since there is no consensus in terms of the factor structure of the scales. Some studies have claimed that the short DAS is a one-factor structure [10,13], while others have shown that a three-factor structure of DAS (hostile gesture, safety-blocking, and arrival-blocking) best fit the data [7,50]. The PCA technique offers an opportunity to explore the DAS factor structure from an exploratory perspective, the results of which would provide important evidence to reconfirm the validity of the three-factor structure. The three-factor structure identified in [7] is presented in the third column of Table 2. Item 11 was not classified into any of the three factors since it was crossly loaded on arrival-blocking and safety-blocking factors.

The PCA process generated three valid factors (Table 2) with an eigenvalue >1 and Cronbach’s α > 0.60. A total of 56.44% variance in the driving anger data was explained by this three-factor solution. This factor structure is highly coincident with the three-factor structure previously proposed by Zhang et al. [7]. Specifically, Factor 1 contained two items (9 and 10) from the original hostile gesture factor, as well as item 11, and was labeled as “hostile gesture”. Factor 2 consisted of seven items from the original arrival-blocking factor and was therefore labeled as “arrival-blocking”. Factor 3 was formed by four items from the original safety-blocking factor and was labeled as “safety-blocking”. As a result, the previous three-factor structure of the 14-item DAS was reconfirmed here using the PCA technique. A mean score on each subscale was computed on the basis of the items within each scale.

### 3.3. Psychometric Properties of the DBQ

The content and mean rating for each item in the DBQ is presented in Table 3. Similar to the analysis on the DAS, the factor structure of the DBQ was investigated with PCA. Three factors, accounted for a total of 42.49% of the variance in DBQ data, were generated (Table 3). Items 6 and 11, with factor loadings of <0.40 on all three factors, were excluded from further analysis. Among the three factors, Factor 1 accounted for 27.69% of the variance in DBQ data and had a good Cronbach’s α of 0.853. Items in this factor were traffic violations involving anger and Factor 1 was therefore named “emotional violation”. Factor 2 explained 9.01% of the variance with an acceptable internal reliability of 0.669. Items in this factor were also traffic violations, but without much emotion involved. They were deliberate deviations from safe driving and therefore Factor 2 was labeled as “deliberate violation”. There were six items in Factor 3 and they accounted for 5.79% of the variance. Factor 3 was labeled as “error” since all items in the category were inappropriate driving behaviors due to drivers’ misjudgments or failures of observations. The Cronbach’s α of this error factor was 0.648, indicating an acceptable internal reliability. A mean score on each DBQ subscale was calculated on the basis of the items within each scale.

### 3.4. The Mediated Model

**Figure 2:**
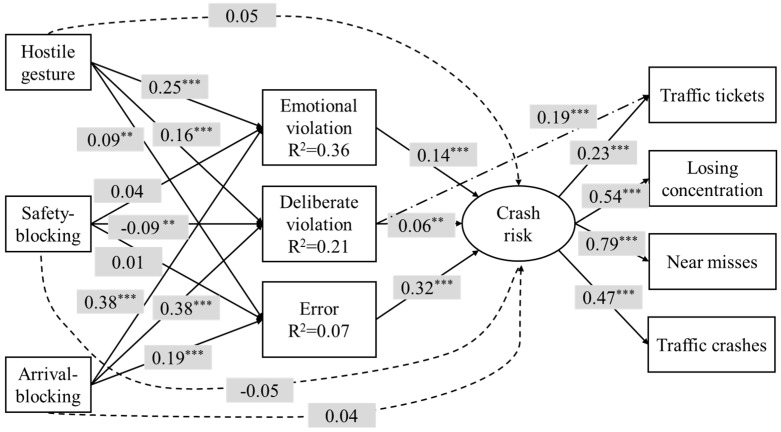
Results of the mediated model. Values on the paths are the standardized path coefficients. The dashed lines represent the direct effects of driving anger on crash risk and the dash-dot line represents the direct effect of deliberate violation on traffic tickets. R^2^ represents the amount of variance the factor is accounted for in the model. ** *p <* 0.01; *** *p* < 0.001.

A mediated model was developed (see Figure 2) in accordance with the framework proposed in Figure 1. Specifically, the three types of driving anger were the distal predictors, the three categories of aberrant driving behaviors were the mediators, and the crash-related condition was the variable being explained. The effects of age, gender, and driving experience on crash involvement were controlled. The proposed model was evaluated using the SEM technique. The SEM results suggested that the model would fit the data better by allowing deliberate violation to have a direct effect on traffic tickets. Therefore, one path between deliberate violation and traffic tickets (represented by the dash-dot line in Figure 2) was added to the model. The goodness of fit of the adjusted model was then evaluated using the SEM technique again. The results showed that the CFI value was 0.959, which was higher than the 0.95 cut-off criterion. The values for RMSEA and SRMSR were 0.055 and 0.041, respectively, both of which were below the maximum allowable value of 0.08. The values of these indices suggested that the adjusted mediated model fit the data very well.

In terms of the confounding effects of demographic variables, it was found that drivers with more driving experience were less likely to get involved in traffic crashes (standardized coefficient *β* = −0.232, *p* < 0.001). No significant effect of age and gender on crash risk was identified. To simplify the presentation of the model, the effects of age, gender, and driving experience on the four crash-related conditions were not shown in the mediated models. Other standardized path coefficients (*β*) are placed on the corresponding paths in Figure 2. Regarding driving anger–aberration associations, it was found that hostile gesture anger and arrival-blocking anger were positively associated with all three types of aberrant driving behaviors. Safety-blocking anger had a significantly negative effect on deliberate violation (*β* = −0.09), but no effect on emotional violation (*β* = 0.04) or error (*β* = 0.01). About 36% of the variance in emotional violation and 21% of the variance in deliberate violations were explained by driving anger. Only 7% of the variance in driving errors was explained. For driving aberration–crash associations, all three types of driving aberrations were positive predictors of crash risk, but with different magnitudes of effect. The error showed the greatest magnitude of effect on crash risk, followed by emotional violation, and deliberate violation showed the smallest magnitude of effect. As was hypothesized, the direct effects (the values on dashed lines in Figure 2) of the three types of driving anger on crash risk were insignificant. These results suggested that hostile gesture and arrival-blocking anger could increase driver crash risk by promoting all three types of driving aberration. On the other hand, safety-blocking anger could decrease crash liability by reducing deliberate violations. In total, 31% of the variance in crash risk was explained in this model. Finally, it was found that deliberate violation showed a positive direct effect on traffic tickets (*β* = 0.19).

A bootstrapping procedure was employed to test the significance of the mediating effect of aberrant driving behaviors. A thousand bootstrap samples were generated according to random sampling from the data set (*n* = 1974). The standardized direct, total indirect, and total effects are summarized in Table 4. The total indirect effect is the sum of the specific indirect effects of driving anger on crash risk via the three types of driving aberration. Arrival–blocking anger was the strongest predictor of crash risk, followed by hostile gesture anger. Both the direct and total indirect effects of safety-blocking anger on crash risk were insignificant.

## 4. Discussion

After the relations between driving anger and crash risk were demonstrated, it was necessary to turn to the explanation and theory testing regarding those relations. This study investigated whether aberrant driving behaviors would mediate the effects of driving anger on crash involvement risk. In general, the results support the conclusion that driving anger influences certain types of aberrant driving behaviors, which further affect the crash liability of drivers. However, the magnitudes of the mediated effects were shown to be dependent on the specific type of driving anger and aberrant driving.

For anger–aberration relationships, about 36% of the variance in emotional violations and 21% of the variance in deliberate violations were explained by driving anger. Only 7% of the variance in driving errors was explained, a result that is not surprising as driving errors are more related to anxiety [51] or stress [52] rather than driving anger. Our results confirmed the findings reported by Zhang et al. [7] that the strengths and directions of anger–aberration relations differed across the three types of driving anger. Consistent with the widely accepted positive anger-aberration relation, it was found that arrival-blocking anger and hostile gesture anger were positive predictors of all three categories of driving aberrations. However, the positive relation did not apply to safety-blocking anger, which was found to be negatively related to deliberate violations and unrelated to emotional violations and errors. This is probably because those who are more intensely angered by safety-threating events may have stronger safety-mindedness and are less likely to violate traffic rules [53].

On the driving aberration–crash risk relationships, it was found that driving errors and emotional violations were more relevant predictors than deliberate violations. This finding is quite reasonable since drivers tend to commit deliberate violations when they are confident in controlling the traffic situation [54]. This means that deliberate violations would be committed only when the crash risk was evaluated to be low by the driver. On the other hand, both emotional violations and errors are behaviors involving no or little logical analysis, which may increase crash risk when adopted on the road. However, this is not the case for one specific crash-related condition—the traffic ticket. The SEM results suggested that deliberate violations had a strong direct effect on traffic tickets. This result is consistent with the definition of the traffic ticket—that it entails violations of traffic laws [55].

On the anger–crash risk relationships, the results of SEM support the proposed mediated model, suggesting that aberrant driving behaviors serve to clarify the nature of the relationship between driving anger and crash liability. These findings support the crash prediction framework proposed by Elander et al. [31], that driving anger is distal while aberrant driving behaviors are proximal factors in predicting traffic crashes. However, dissimilar to previous mediated models, where overall driving anger and aberrant driving behaviors were used [32,33], this is the first study that has applied the subscales of driving anger and aberrant driving behaviors in the mediated model. The results showed that the working mechanisms of hostile gesture anger and arrival-blocking anger in influencing crash risk were quite similar. Both of them increase the probability of drivers committing aberrant driving behaviors, which, in turn, increase the crash risk. However, the magnitudes of their effects on crash risk differed, with arrival-blocking anger showing stronger effects than hostile gesture anger. In terms of safety-blocking anger, there were indirect negative effects on crash risk via the mediator of deliberate violations. The results, that different types of driving anger have dissimilar effects on road crash risk, suggest that the anger–crash relationships previously established using an overall driving anger score [12,13] have masked the real effects at the subscale levels.

The mediated models established in the present study provide a better understanding of the role of driving anger in the causation of road traffic crashes, which should contribute to developing effective countermeasures. First, our results suggest that the serious traffic congestion problem in China may contribute to the high road crash rate among Chinese drivers. When stuck in traffic, arrival-blocking anger would be provoked, making drivers more likely to commit driving aberrations and further increasing the crash risk. It is expected that solutions aimed at relieving traffic congestion have the potential to reduce the crash rate in China. Second, our results indicate that countermeasures in treating drivers with high hostile gesture and arrival-blocking anger would be effective to reduce crash risk. Currently, no training or treatment programs on high-anger drivers have yet been developed in China. It is suggested that future research should start to develop effective anger treatment strategies and evaluate whether these strategies can improve road safety in China. Third, since the effects of driving anger on crash risk are fully mediated by aberrant driving behaviors, it is possible to improve road safety with driving aberration intervention. In road safety literature, education campaigns and law enforcement have proven to be effective in reducing deliberate violations [2,56]. Taking China as an example, it has been found that anti-speeding devices and heavier traffic fines have successfully reduced speeding on the road [2]. According to Lajunen et al. [53], emotional violations can be reduced by improving drivers’ tolerance of frustration and their management of anger. In terms of decreasing driving errors, training programs and driver assistance systems (e.g., the autonomous cruise control system) have proven to be effective [57,58].

One major limitation of this study is its reliance on self-reported measures, the data from which may suffer from social desirability bias. That is, aberrant driving behaviors may be underreported due to the deliberate tendency of respondents to give answers in a manner that will be viewed favorably to others. However, some researchers have found that self-reported driving aberrations were only slightly affected by social desirability bias [59,60]. Moreover, the anonymity of the Internet survey may have partially offset such bias. Another problem is that self-reported crash-related data, especially for near misses, may suffer from recall bias [61] and might not be reliable if respondents do not fully understand the question, although the questionnaire provided some definitions [62]. Future studies may be undertaken to test the mediated model by means of more objective measures of crash risk, though it should be mentioned that a recent study comparing self-reported and police-recorded traffic crashes found them to be strongly correlated [63]. Also, epidemiological data have indicated that crash risk is the highest within the first year of licensure and then improves dramatically [64]. Therefore, setting the lowest level of driving experience to be less than three years might have not captured the effect of driving experience on crash risk. Finally, the sample size of the survey might not be representative of the general driving population in China. For instance, the majority of the respondents were male; therefore, one should be cautious in generalizing the findings.

## 5. Conclusions

This study demonstrated that aberrant driving behaviors fully mediate the effects of driving anger on crash risk. Importantly, the magnitude and significance of the associations in the mediated models depend on the specific type of driving anger and aberrant driving behaviors. These findings contribute to road safety research by providing a deeper understanding of the role of driving anger and aberrant driving in the causation of road traffic crashes. The results of the mediated model suggest that crash risk can be decreased either by relieving hostile gesture anger and arrival-blocking anger or by reducing driving aberrations. Therefore, this study is useful in guiding the development of countermeasures aimed at reducing road traffic crashes in China.

## Figures and Tables

**Table 1:** Descriptive statistics for the DAS, DBQ, and crash-related conditions.

Variables	Mean	Standard Deviation
DAS	2.45	0.69
DBQ	2.19	0.73
Traffic tickets	0.80	0.95
Losing concentration	0.91	1.12
Near misses	0.53	0.72
Traffic crashes	0.23	0.45

**Table 2:** Mean and standard deviation (SD) of each DAS item and the results (number of factors determined, factor loading, etc.) of PCA.

DAS Items	Scenarios	Original Factor Category ^a^	Mean (*SD*)	Factor1 Hostile Gesture (40.19%) α = 0.797	Factor2 Arrival-Blocking (9.17%) α = 0.817	Factor3 Safety-Blocking (7.08%) α = 0.688
9	Someone makes an obscene gesture toward you about your driving	HS	3.25 (1.29)	0.836		
10	Someone honks at you about your driving	HS	2.90 (1.20)	0.794		
11	A bicyclist is riding in the middle of the lane and is slowing traffic	×	2.96 (1.18)	0.606		
14	You are driving behind a large truck and you cannot see around it	AB	1.95 (1.02)		0.732	
5	You pass a radar speed trap	AB	1.43 (0.79)		0.722	
12	A police officer pulls you over	AB	1.63 (0.90)		0.706	
13	A truck kicks up sand or gravel on the car you are driving	AB	2.64 (1.20)		0.549	
8	You are stuck in a traffic jam	AB	2.28 (1.05)		0.538	
6	Someone speeds up when you try to pass him/her	AB	2.47 (1.10)		0.491	
7	Someone is slow in parking and is holding up traffic	AB	2.32 (1.05)		0.478	
4	Someone runs a red light or stop sign	SB	2.24 (1.22)			0.750
1	Someone is weaving in and out of traffic	SB	2.18 (0.97)			0.633
3	Someone backs right out in front of you without looking	SB	2.95 (1.16)			0.603
2	A slow vehicle on a mountain road will not pull over and let people by	SB	3.16 (1.16)			0.520
Mean (*SD*)			2.45 (0.69)	3.04 (1.03)	2.63 (0.81)	3.10 (0.71)

^a^: The factor category was based on the study by Zhang et al. [7]. ×: Item 11 has been excluded from any of the three factors in previous study [7]. HS: hostile gesture; AB: arrival-blocking; SB: safety-blocking. Note: The value inside the bracket under a factor is the percentage of variance in driving anger data explained by that factor. All the Cronbach’s α values for the three factors are larger than 0.60, indicating an acceptable internal consistency.

**Table 3:** Mean and standard deviation (SD) of each DBQ item and the results (number of factors determined, factor loading, etc.) of PCA.

DBQ Items	Aberrant Driving Behaviors	Mean (*SD*)	Factor1 Emotional Violation (27.69%) α = 0.853	Factor2 Deliberate Violation (9.01%) α = 0.669	Factor3 Error (5.79%) α = 0.648
13	Warn a slow car in front to drive faster	2.60 (1.07)	0.768		
22	Give chase when angered by another driver	2.04 (0.96)	0.760		
17	Sound horn to indicate annoyance to another driver	2.63 (1.10)	0.723		
15	Aversion to other road users and indicate hostility to them	2.00 (1.09)	0.713		
4	Drive fast when in bad mood	2.67 (1.18)	0.637		
12	Drive fast to pass a yellow light turning to red	2.85 (1.13)	0.540		
18	Unknowingly speeding	2.40 (1.01)	0.530		
9	Tailgating the vehicle that angered you	2.32 (1.02)	0.508		
14	Do not give way to cyclists when turning right	1.70 (0.84)	0.461		
3	Driving wrong way on opposite lanes	1.55 (0.79)		0.679	
8	Disregard the traffic light	1.51 (0.82)		0.650	
1	Drive under the influence of alcohol	1.28 (0.57)		0.551	
16	Use a non-motor lane	1.84 (0.91)		0.539	
5	Overtake on the right side	2.78 (1.00)		0.483	
20	Fail to notice “left-turn-forbidden” signs	2.27 (0.71)			0.714
19	Distracted, have to brake hard	1.97 (0.65)			0.605
2	Get into the wrong lane	2.41 (0.72)			0.602
21	Forget which gear	1.61 (0.72)			0.597
10	Fail to notice a pedestrian crossing	1.59 (0.67)			0.484
7	Distracted, misjudge interval and narrowly miss collision	1.17 (0.42)			0.448
Mean (*SD*)		2.05 (0.47)	2.36 (0.70)	1.79 (0.55)	1.83 (0.39)
6	Fail to notice “give-way” signs	2.41 (1.19)	0.235	0.358	0.345
11	Stop on road where stopping/parking is not allowed	2.12 (1.00)	0.142	0.329	0.368

Note: The value inside the bracket under a factor is the percentage of variance in DBQ data explained by that factor. All the Cronbach’s α values for the three factors are larger than 0.60, indicating an acceptable internal consistency. Items 6 and 11 were excluded from further analysis due to their low factor loading (<0.40) on all three factors.

**Table 4:** The standardized direct, indirect, and total effects of each type of driving anger on crash risk.

Effect Types	Hostile Gesture	Safety-Blocking	Arrival-Blocking
Indirect effect	0.073 **	0.004	0.137 **
Direct effect	0.054	−0.049	0.037
Total effect	0.127 **	−0.045	0.174 **

** *p* < 0.01.
